# Mitogen-Activated Protein Kinase p38b Interaction with Delta Class Glutathione Transferases from the Fruit Fly, *Drosophila melanogaster*

**DOI:** 10.1673/031.012.10701

**Published:** 2012-09-05

**Authors:** Jeerang Wongtrakul, Suchada Sukittikul, Chonticha Saisawang, Albert J. Ketterman

**Affiliations:** ^1^ Research Institute for Health Sciences (RIHES), Chiang Mai University, P.O.BOX 80 CMU, Chiang Mai, Thailand 50200; ^2^ Institute of Molecular Biosciences, Mahidol University, Nakhon Pathom, Thailand 73170

**Keywords:** MAP kinase pathway, signal modulation, substrate specificity ATF2, jun

## Abstract

Glutathione transferases (GSTs) are a family of multifunctional enzymes involved in xenobiotic biotransformation, drug metabolism, and protection against oxidative damage. The p38b mitogen-activated protein kinase is involved in cellular stress response. This study screened interactions between *Drosophila melanogaster* Meigen (Diptera: Drosophilidae) Delta class glutathione transferases (DmGSTs) and the *D. melanogaster* p38b MAPK. Therefore, 12 DmGSTs and p38b kinase were obtained as recombinant proteins. The study showed that DmGSTD8 and DmGSTD11b significantly increased p38b activity toward ATF2 and jun, which are transcription factor substrates. DmGSTD3 and DmGSTD5 moderately increased p38b activity for jun. In addition, GST activity in the presence of p38b was also measured. It was found that p38b affected substrate specificity toward CDNB (1-chloro-2,4-dinitrobenzene) and DCNB (1,2-dichloro-4-nitrobenzene) of several GST isoforms, i.e., DmGSTD2, DmGSTD5, DmGSTD8, and DmGSTD11b. The interaction of a GST and p38b can affect the substrate specificity of either enzyme, which suggests induced conformational changes affecting catalysis. Similar interactions do not occur for all the Delta enzymes and p38b, which suggests that these interactions could be specific.

## Introduction

Glutathione transferases (GSTs), phase II metabolizing enzymes, comprise a large family of enzymes that catalyze the conjugation of tripeptide glutathione (GSH) to a variety of electrophiles. These substrates include lipid hydroperoxides generated by oxidative stress, as well as xenobiotics, allowing for their inactivation and more rapid excretion (Hayes et al. 1995; Armstrong 1997; Sheehan et al. 2001; Awasthi et al. 2004). In mammals, GSTs are categorized into seven distinct classes based on the amino acid sequence identity, physical structure of the genes (intron number and position), and immunoreactivity properties: Alpha, Mu, Pi, Sigma, Theta, Zeta, and Omega, (Hayes et al. 2005; Frova 2006). Apart from cellular detoxification roles, GSTs also act as transport and ligand binding proteins. There are several reports that GSTs can interact and modulate proteins in mitogen-activated protein kinase (MAPK) pathways, i.e., Jun N-terminal kinase (JNK/SAPK), TRAF2, and ASK1 (Adler et al. 1999; Cho et al. 2001; Wu et al. 2006; Zhao et al. 2006; Laborde 2010).

Briefly, cellular behavior in response to extracellular stimuli is mediated via intracellular signaling pathways such as the MAPK pathways. At least three families have been characterized: extra-cellular signal-regulated kinase, JNK/SAPK, and p38 MAPK. Each group of MAPKs can be stimulated by a separate protein kinase cascade that includes the sequential activation of a specific MAPK kinase kinase and a MAPK kinase, which in turn phosphorylate and activate their downstream MAPKs. The p38 families are of particular interest, as the activation of p38 relays extracellular stimuli to transcription factors, such as ATF2, jun, p53, etc., as well as protein kinases. The p38 pathway, therefore, regulates gene expression, apoptosis, differentiation, proliferation, and especially inflammation, which is a crucial factor in the pathogenesis of several different human diseases, including neural diseases, making the pathway a potential target for development of anti-inflammatory therapeutics (Zarubin et al. 2005; Aracena-Parks et al. 2006; Coulthard et al. 2009; Yong et al. 2009; Yasuda et al. 2011). In addition, knowledge of GST modulation of proteins in MAPK signaling would be an alternative target in therapy. However, the possibility of GSTs modulating the p38 pathway remains unknown. Evidence based on GST and JNK studies suggest that GSTs could be involved in regulation of the p38 signaling pathway. For example, GSTs have been shown to be involved in the H_2_O_2_-activated p38 signaling pathway. It was found that 129 vascular smooth muscle cells, which contain higher levels of GSTM1 compared with C57BL/6 vascular smooth muscle cells, demonstrated significantly less p38 phosphorylation compared with C57 after H_2_O_2_ exposure (Yang et al. 2009). Knockdown of GSTM1 by siRNA resulted in increased p38 kinase phosphorylation. Similar results were found in RAW264.7 cells transient and stable transfected with GSTP1 (Xue et al. 2005). GSTP1 demonstrated an inhibitory effect on mediating LPS-induced p38 MAPKs activation. GST also has been shown to inhibit the activation of the ASK1-p38 signaling pathway (Dorion et al. 2002). In stress conditions, such as heat shock or exposure to H_2_O_2_, GSTM1 is thought to be released from ASK1, leading to activation of ASK1 and its downstream targets. However, a different role for GST interaction with p38 was observed in a 3T3 cell line. This cell line had low basal level of GST pi. Inducing higher expression of GST pi, using a time-dependent gradual increase in expression, showed a substantial increase in p38 phosphorylation. Moreover, the effect of GST pi expression on stress kinases following exposure to H_2_O_2_ treatment increased p38, extra-cellular signal-regulated kinase, and IkB kinase activities. GST pi expression reduced the degree of H_2_O_2_-induced JNK phosphorylation. These observations suggested a coordinated regulation of stress kinases after H_2_O_2_ treatment of GST pi expressing cells (Yin et al. 2000).

The p38 MAPK studies in mammals are complex; there are many isoforms of p38, so pathway interpretation is difficult. *Drosophila melanogaster* Meigen (Diptera: Drosophilidae) was chosen for this study because the components of the stress activated protein kinase pathways are structurally and functionally conserved, and not as complicated as in mammals. For example, there are only two isoforms in *D. melanogaster*, p38a and p38b (Han et al. 1998). p38b recombinant protein was used in our study, because p38a mutant flies do not show effects in their longevity or developmental consequences (Craig et al. 2004). Dominant negative p38b affects the wing imaginal disc and the process of wing morphogenesis (Adachi-Yamada et al. 1999). In addition, genetic ablation of p38b resulted in small flies consisting of small cells. The p38 mutants are nutrition sensitive; lownutrient food accentuates the small-organism phenotypes, including partial lethality (Cully et al. 2010). In adult flies, p38b has a role in the balance between intestinal stem cell proliferation and appropriate differentiation in adult midgut (Park et al. 2009). In addition, the sensitivity to microbial infection was significantly higher in p38b mutant flies, which suggested that the p38 pathway contributed to *D. melanogaster* host defense against microbial infection (Chen et al. 2010).

We are interested in elucidating the interaction of GST with the *D. melanogaster* p38b kinase. An *in vitro* kinase assay was used to measure p38b activity in the presence and absence of GST in order to determine substrate specificity changes toward two kinase substrates, ATF2 and jun. Kinase effects on GST activity toward two classical GST substrates, CDNB (1-chloro-2,4dinitrobenzene) and DCNB (1,2-dichloro-4-nitrobenzene) were also studied. Our data showed interactions occurred between several GST isoforms and p38b kinase.

## Materials and Methods

### Cloning of *D. melanogaster* DmGSTs, p38b, and ATF2

For GST cloning, mRNAs were isolated from a *D. melanogaster* S2 cell line, whereas for the MAPK pathway proteins, the mRNAs were isolated from the *D. melanogaster* adult fly by using TRIzol™ LS reagent (Gibco BRL, http://www.lifetechnologies.com/), as described in the manufacturer's instructions. The first strand cDNA was synthesized by using SUPERSCRIPT™ II Rnase H Reverse Transcriptase (Gibco BRL), according to the manufacturer's protocol. The sets of oligonucleotide primers for *D. melanogaster* proteins were designed according to specific 5′ and 3′ sequences of genes obtained from the Genbank database. The recombinant clones were verified by full-length sequencing in both directions. *Escherichia coli* BL21 (DE3)pLysS recombinant clones were used for protein expression.

### Purification of *D. melanogaster* DmGSTs, p38b,ATF2, and jun

Protein purification of DmGSTD1, DmGSTD2, DmGSTD7, DmGSTD9, DmGSTD11a, and DmGSTD11b was performed using GSTrap affinity chromatography, according to the manufacturer's instruction. For the purification of DmGSTD3, DmGSTD5, DmGSTD6, DmGSTD8, and DmGSTD10, first a HiTrap Q XL anion exchanger, then a HiTrap phenyl Sepharose column, were employed. The conditions for the first column were 50 mM Tris pH ranging from 7.5 to 8.5, and salt elution from 50 to 300 mM, depending on the GST. The enzymes eluted from the second column with a decreasing salt gradient in 50 mM Tris pH 8.0. For the purification of DmGSTD4, the first column was HiTrap Q-HP, and isocratic elution with 50 mM Tris pH 8.0. The elution was collected, concentrated, and desalted with HiTrap desalting column using 50 mM phosphate buffer pH 6.5 for application to the second column, a HiTrap SP-XL. DmGSTD4 was applied, and the flow through fraction with the enzyme was collected.

The purification of p38b was performed as previously described (Bukhtiyarova et al. 2004). For ATF2, a HiTrap Chelating HP (GE Healthcare, www.gehealthcare.com) charged with NiCl_2_ was used according to the manufacturer's instruction. The expression and purification of jun was carried out as previously described (Udomsinprasert et al. 2004). The purified proteins were stored in 50% glycerol, 10 mM DTT, 50 mM phosphate buffer pH 6.5 for GSTs, or 50 mM Tris-HCl pH 8.0 for kinase proteins at -20° C.

### Protein kinase assays

The p38b present in each of the different *D. melanogaster* GST isoforms was assayed for activity using ATF2 or jun as substrate. Kinase activity assays were performed using the ADP Quest Assay (DiscoveRx, http://www.discoverx.com/). Kinase activity was monitored using a fluorescence plate reader (Synergy™ HT, BIO-TEK®, http://www.biotek.com/) operating in kinetic mode (530 nm excitation and 590 nm emission). All proteins were desalted to remove DTT and glycerol before performing the kinase assay. Briefly, p38b (4µg) was combined using 1:1 molar ratio of GST and 100 µM ATP in ADP assay buffer. The solution was incubated for 3 minutes at room temperature, before addition of 10 µg ATF2/jun, reagent A, and reagent B respectively. All reactions were performed in a final volume of 100 µl. Fluorescence intensity values were plotted against time to obtain a slope. The correlation between fluorescence intensity and time was linear. The background reaction rate, resulting from ADP degradation of the ATP stock solution, was measured in a reaction lacking enzyme. Enzyme activities were obtained by subtracting the slope of background from the experimental sets. All experiments were performed in triplicate, and the activities were calculated using Graphpad Prism 4.0 (GraphPad Software, Inc., http://www.graphpad.com/).

### GST activity assays and effect of p38b on GST activity

GST activities were measured spectrophotometrically (Spectra MR Microplate spectrophotometer, DYNEX technologies, http://www.dynextechnologies.com/). All measurements were performed between 25– 27° C in 0.1 M potassium phosphate buffer (pH 6.5 or 7.5). The activity was measured by the conjugation of GSH with the hydrophobic substrates CDNB, DCNB, PNBC (p-nitrobenzyl chloride), and PNPB (p-nitrophenethyl bromide) (Habig et al. 1974). The effect of p38b kinase on GST activity was examined by incubating GSTs and p38b using 1:1 molar ratio at room temperature for 5 minutes. Then GST activity toward CDNB and DCNB was measured in the presence and absence of p38b. The percent change was calculated based on the activity of each GST as 100% in the absence of p38b.

**Table 1.**
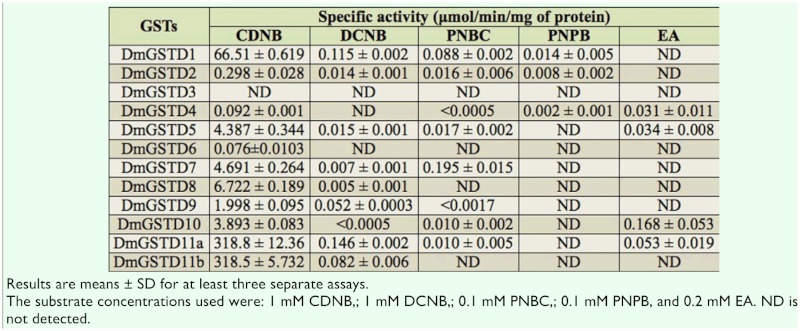
Specific activity of *Drosophila melanogaster* glutathione transferasesGSTs using various xenobiotic substrates.

## Results

### Modulation of p38b kinase activity by the presence of DmGSTs

**Figure 1.**
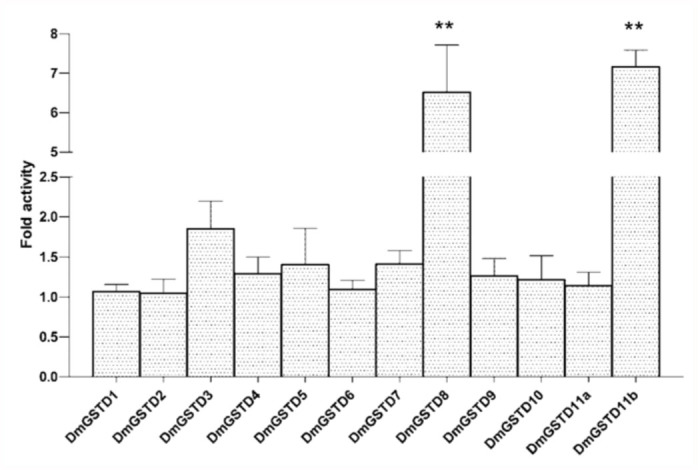
Delta class glutathione transferases (DmGSTs) modulate p38b kinase activity toward the substrate ATF2 in *Drosophila melanogaster*. The histogram shows changes in fold activity the DmGSTs have on p38b phosphorylation rates compared to the reaction without DmGSTs. The experiments were performed in triplicate. One-way ANOVA with Dunnett's multiple comparison test was performed with kinase reaction lacking DmGST as a control; statistical significance is shown by ** for *p* < 0.01. High quality figures are available online.

**Figure 2.**
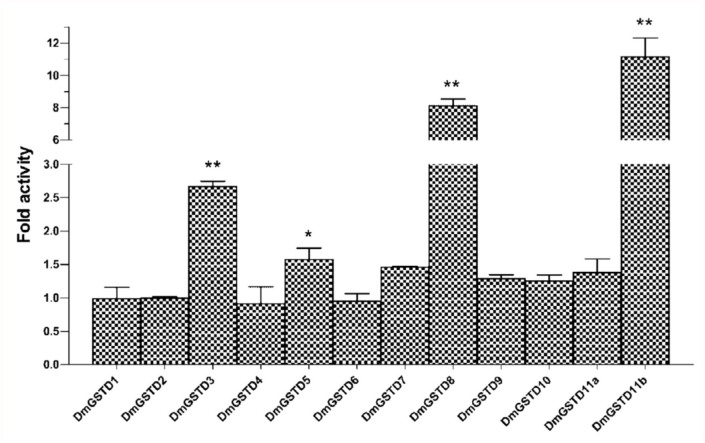
Delta class glutathione transferases (DmGSTs) modulate p38b kinase activity toward the substrate jun in *Drosophila melanogaster.The* histogram shows changes in fold activity the DmGSTs have on p38b phosphorylation rates compared to the reaction without DmGSTs. The experiments were performed in triplicate. One-way ANOVA with Dunnett's multiple comparison test was performed with kinase reaction lacking DmGST as a control; statistical significance is shown by * for *p* <0.05 and ** for *p* < 0.01. High quality figures are available online.

To investigate whether *D. melanogaster* GSTs are capable of modulating the p38b activity, *in vitro* kinase activity was measured in the presence of the GSTs. Over all, DmGSTD1, DmGSTD2, DmGSTD4, DmGSTD6, DmGSTD7, DmGSTD9, DmGSTD10, and DmGSTD11a had no effect on kinase activity for ATF2 (Figure 1). It was found that DmGSTD3 and DmGSTD5 slightly activated p38b, approximately 1.8 and 1.4 fold respectively, whereas DmGSTD8 and DmGSTD11b significantly increased p38b activity 6.5 and 7.1 fold respectively. Then, to determine whether p38b substrate specificity changes upon interaction with GST, jun was used as the kinase substrate. It was found that the effects of individual GSTs on p38b phosphorylation of jun were similar to ATF2 (Figure 2). Moderate effects on phosphorylation by p38b were observed with DmGSTD3 and DmGSTD5. Both GSTs significantly increased p38b activity 2.6 and 1.6 fold respectively for jun. DmGSTD8 and DmGSTD11b showed the greatest increase in p38b kinase activity,approximately 8.1 and 10 fold respectively. Collectively, these data show that specific GSTs are capable of interacting with and modulating p38b MAPK. Several GSTs appear to increase the kinase activity for ATF2 and jun phosphorylation.

### GST substrate specificity

An initial screen of the activities of the 12 GSTs with 5 xenobiotic substrates was performed to identify substrates for use in monitoring enzyme changes that may occur in the presence of p38b MAPK. The substrates tested are classical GST substrates: CDNB (a general GST substrate), DCNB, 4-nitrophenethyl bromide, 4- nitrobenzylchloride, and ethacrynic acid (Mannervik et al. 1988; Hayes et al. 1995). The results are presented in Table 1. DmGST activities toward these substrates were different among the isoenzymes. CDNB, DCNB, and 4-nitrobenzylchloride were the best substrates among the tested GSTs. DmGSTD11a and DmGSTD11b isoforms had the greatest activity toward CDNB. In addition DmGSTD11a had the greatest DCNB activity among the tested Delta GSTs. In contrast, DmGSTD11b had DCNB activity only 0.5 fold of DmGSTD11a. DmGSTD11b had no activity toward 4-nitrophenethyl bromide, 4-nitrobenzylchloride, and ethacrynic acid. DmGSTD7 had the greatest activity toward 4-nitrobenzylchloride. 4nitrophenethyl bromide conjugating activity was shown only by DmGSTD1, DmGSTD2, and DmGSTD4, although DmGSTD1 possessed 1.78 and 5.8 fold greater activity than DmGSTD2 and DmGSTD4, respectively. Unsurprisingly, DmGSTD3 was not active toward any of the five tested substrates, most likely because it is missing 15 residues at the N-terminus, including the important catalytic serine. For pi class substrate ethacrynic acid, DmGSTD10 displayed the highest EA-conjugating activity.

**Figure 3.**
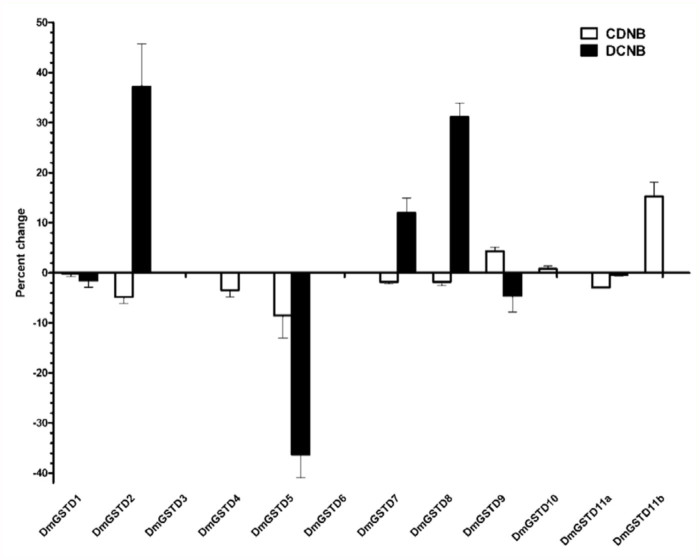
p38b changes the substrate specificities of Delta class glutathione transferases (DmGSTs) in *Drosophila melanogaster*. DMGSTs and p38b were incubated in 1 : 1 molar ratio for five minutes at room temperature. DmGST activity was measured using CDNB and DCNB as substrates. The percentage change in DmGST activity was calculated by comparing the reactions in the presence and absence of p38b. Results shown are representative of triplicate independent experiments. High quality figures are available online.

DmGST substrate specificity changes upon co-incubation with p38b are shown in Figure 3. Interestingly, incubation with p38b increased DmGSTD2 activity toward DCNB approximately 37%, whereas it slightly decreased on testing with CDNB substrate. DmGSTD5 activity toward CDNB was decreased approximately 10%. A more severe reduction of about 40% was observed when it was incubated with p38b and tested with DCNB substrate. DmGSTD7 and DmGSTD8 activities toward DCNB were increased 10% and 30% respectively. Unfortunately, DmGSTD6 was unstable, as incubation for five minutes at room temperature affected the enzyme stability, resulting in total loss of GST activity.

## Discussion

In this study, we report the non-catalytic role of *D. melanogaster* Delta class GSTs in the modulation of p38 MAPK signaling pathway, as well as the role of p38b kinase protein in affecting substrate specificity of GSTs. There are two p38 genes in *D. melanogaster*, p38a and p38b. The amino acid sequence of p38b is 75% identical to that of p38a (Han et al. 1998). The p38b protein is highly conserved compared to the four human p38 isoforms, with a 55–73% amino acid identity and 72–87% sequence similarity. *D. melanogaster* GSTs are categorized into 6 classes (Tu et al. 2005), with the Delta class being the most studied. There are 12 protein isoforms of Delta class GST. The enzymes share 30–91% amino acid identity, and 47–91% sequence similarity.

DmGSTD3 lacks 15 amino acids at the N-terminus, including the serine residue that is involved in catalysis. Our results suggest a role for DmGSTD3 in p38b MAPK signaling, as DmGSTD3 interacted with p38b and increased jun phosphorylation rates 2.6 fold (Figure 2). As DmGSTD3 is inactive, this finding indicates a non-catalytic role of DmGSTD3 in the modulation of p38b. This was also reported for GSTM1-1 (Y6F), an inactive mutant of mu class GST. The mutant was released from ASK1, the upstream activator of p38, during heat shock, which resulted in p38 activation (Dorion et al. 2002). In adult flies, treatment with the herbicide paraquot led to a significant increase in the relative amount of DmGSTD3, approximately 1.3 fold (Alias et al. 2007). Paraquot is a potent herbicide that produces ROS during a cyclic reaction with oxygen. The ROS then damages the cell and results in lipid peroxidation products. Although DmGSTD3 appears to lack enzymatic activity, there is accumulating evidence that suggests DmGSTD3 plays a role in oxidative stress response. This is possibly through the modulation of p38 MAP kinase pathway, which is known to be involved in stress response.

DmGSTD2 is of interest because it lacks the serine that directly interacts with the GSH thiol. DmGSTD2, instead, has glycine in this position, although it still possesses detectable CDNB activity. Although DmGSTD2 did not appear to affect p38b activity, it was found that p38b could interact and increase DmGSTD2 activity 37% toward DCNB (Figure 3). A previous report showed DmGSTD2 had 4-HNE conjugating activity, suggesting a physiological function (Sawicki et al. 2003). 4-HNE is a lipid peroxidation product that can also affect cell signaling pathways (Awasthi et al. 2004). In addition, DmGSTD2 also played a role in the defense mechanism against oxidative stress. A previous DmGSTD2 study showed phenobarbital treatment of flies led to increases of approximately 1.7 fold in the relative amount of DmGSTD2 (Alias et al. 2007). It has been reported that DmGSTD2 has peroxidase activity toward H_2_O_2_ and t-butyl hydroperoxide (Tang et al. 1994; Sawicki et al. 2003). Therefore, DmGSTD2 appears to have roles in 4-HNE metabolism, and defense against oxidative stress.

DmGSTD8 interacted with p38b and increased p38b phosphorylation toward both ATF2 and jun 6 fold and 8 fold respectively. Moreover p38b increased DCNB activity of DmGSTD8 approximately 30%, corroborating the interaction between the two proteins. Although this GST had no 4-HNE conjugating activity (Sawicki et al. 2003), it possessed peroxidase activity against cumene hydroperoxide. These data suggest DmGSTD8 may have multiple functions in oxidative stress response (Lee et al. 1995).

Of the GSTs in this study, DmGSTD5 showed the greatest similarity to DmGSTD2 with an amino acid identity of 78% and a similarity of 91%. In our study, p38b interacted with both DmGSTD2 and DmGSTD5, and slightly reduced CDNB activity of DmGSTD2 (approximately 5%) and DmGSTD5 (approximately 8%). Interestingly, p38b increased DmGSTD2 activity toward DNCB 37%, but the p38b interaction decreased DmGSTD5 activity approximately 36%. However, DmGSTD5 affected p38b kinase activity by increasing the phosphorylation toward jun substrate approximately 1.5 fold, whereas DmGSTD2 did not change kinase activity of p38b. These data suggest that the GST-p38b kinase interaction is specific, and has a different impact on each protein.

DmGSTD11b, which has the least similarity compared to other Delta GSTs (only 30–36% amino acid identity), significantly increased the ability of p38b to phosphorylate ATF2 and jun substrates. DmGSTD11b, compared to the DmGSTD11a isoform, possesses a 21 amino acid extension at the N-terminus, resulting in the differences between the two alternatively spliced proteins. Both jun and ATF2 are components of the transcription factor AP-1, and these substrate phosphorylation rates are enhanced by DmGSTD11b. This suggests GSTs may self-regulate their own expression, as several GST genes have been shown to possess AP-1 regulatory elements (Hayes et al. 1995; Ding et al. 2005; Hayes et al. 2005).

At present, only one study reports a GST role in modulating the p38 pathway by interaction with its upstream activator ASK1 (Dorion et al. 2002). It was found that heat shock led to the dissociation of GSTM1–1 from ASK1, and over expression of GSTM1–1 inhibited in a dose-dependent manner heat shock induced activation of p38. Our study is the first report of a direct interaction between GST and p38b that activates p38b activity. Although, a similar observation was reported previously for a GST that increased the phosphorylation of jun by JNK MAP kinase (Udomsinprasert et al. 2004). However, unlike previous reports, in our study we observed that none of the GSTs inhibited the kinase. Furthermore, either no effect on kinase activity or an activation of up to 8 to 10 fold was observed. Substrate specificity and phosphorylation efficiency of p38 MAPK signaling depends on the interactions of the docking domain of the downstream target consisting of the basic LXL motif, as well as hydrophobic residues, and the p38 binding motif consisting of CD and ED domains on the p38 surface (Barsyte-Lovejoy et al. 2002; Weston et al. 2002). Whether GSTs interact through these domains remains to be elucidated. In this screening study, the DmGSTs were recombinant proteins studied in an *in vitro* system; therefore, an *in vivo* study should be performed to confirm the observed effects are relevant in a cellular context. However, the interaction between GSTs and p38b kinase appears to result in conformational changes that can affect substrate specificity in a manner distinctive for each protein.

## Abbreviations

DmGSTs,: delta class glutathione transferases;
GSH,: tripeptide glutathione;
GST(s),: glutathione transferase(s);
JNK/SAPK,: Jun N-terminal kinase;
MAPK,: mitogen-activated protein kinase
